# The risk factors and prognosis of delayed perihematomal edema in patients with spontaneous intracerebral hemorrhage

**DOI:** 10.1111/cns.13219

**Published:** 2019-09-22

**Authors:** Wen‐jie Peng, Qian Li, Jin‐hua Tang, Cesar Reis, Camila Araujo, Rui Feng, Ming‐hao Yuan, Lin‐yan Jin, Ya‐li Cheng, Yan‐jie Jia, Ye‐tao Luo, John Zhang, Jun Yang

**Affiliations:** ^1^ Department of Neurology The First Affiliated Hospital of Chongqing Medical University Chongqing China; ^2^ Department of Pediatrics The Third Affiliated Hospital & Field Surgery Institution Army Medical University Chongqing China; ^3^ Department of Physiology and Pharmacology Loma Linda University School of Medicine Loma Linda CA USA; ^4^ Department of Neurology The First Affiliated Hospital of Zhengzhou University Zhengzhou China; ^5^ Department of Biostatistics School of Public Health and Management Chongqing Medical University Chongqing China

**Keywords:** delayed perihematomal edema, prognosis, risk factors, spontaneous intracerebral hemorrhage

## Abstract

**Purpose:**

We hypothesize delayed perihematomal edema (DHE) leads to secondary injury after spontaneous intracerebral hemorrhage (sICH) with a poor prognosis. Hence, we need to investigate the risk factors of DHE and identify whether DHE will predict the poor outcome of sICH.

**Methods:**

We retrospectively recruited 121 patients with sICH admitted to the Department of Neurology from January 2014 to August 2018. After dividing all these patients into DHE group and non‐DHE group, we analyzed the potential risk factors and outcome of DHE using a multivariate logistic regression model.

**Results:**

We conclude DHE after sICH associates with age, hospitalization time, hematoma shape, blood pressure upon admission, alcohol consumption, blood sodium level, and baseline hematoma volume within 24 hours after symptom onset, among which differences were statistically significant (*P* < .05). Logistic regression analysis finally identified that age (OR = 0.958, 95% CI = 0.923‐0.995) and the baseline hematoma volume (OR = 1.161, 95% CI = 1.089‐1.238) were the most significant risk factors for DHE, and moreover, the DHE (OR = 3.062, 95% CI = 1.196‐7.839) was also a risk factor for poor prognosis in sICH patients.

**Conclusion:**

We suggest DHE is a clinical predictor of secondary injury following sICH and poor prognosis. In addition, age and baseline hematoma volume are considered significant high‐risk factors for DHE in patients with sICH.

## 1 INTRODUCTION

Spontaneous intracerebral hemorrhage (sICH) is a generally acknowledged disease that accounts for 10%‐15% of all strokes, with high disability and mortality rates in the short term and long term.^1^, ^2^ Growing studies focus on a variety of treatments such as controlling blood pressure, hemostatic therapy, or removing the hematoma through surgery to improve the functional outcome and reduce the mortality rate. However unfortunately, in addition to the hematoma itself, the secondary hematoma expansion and perihematomal edema (PHE) greatly affect the prognosis. It is currently understood that the development of PHE is a responsible factor for secondary injury after sICH, which usually leads to high cranial pressure, neurological deterioration, and even hernia leading to the extremely high mortality and disability rate, significantly relating to the course of disease.^3^ A recent review article summarized that the PHE formation after sICH is complex and still quite controversial. It is known to occur hours, days, and even weeks following sICH onset,^4^ and it is associated with direct hematoma compression on the surrounding tissues, hematoma toxicity, oxidative stress, and inflammation.^5^, ^6^ It is generally recognized that the disruption of vessels will rapidly cause a hematoma and vasogenic edema in the super‐acute stage of ICH. Except the hematoma occupying effect, the production of thrombin and activation of blood coagulation cascade contributes to a slow progressive edema growth, approximately peaking on the seventh day after ICH.^5^, ^7^, ^8^, ^9^ On the other hand, the breakdown of the blood brain barrier, inflammatory cascades, heme, and iron load secondary to red cell dissolution as well as hemoglobin degradation will give rise to or accelerate PHE, but PHE will ameliorate or disappear with the alleviation of hematoma.^9^, ^10^, ^11^ In our clinical work, we founded there are some patients admitted on hospital due to headache, vomiting, visual disturbance, or other symptoms caused by increased intracranial pressure, which is ascribed to the massive PHE happening on the convalescence after sICH. Many studies only mentioned the possible formation of delayed perihematomal edema, but did not give us a explicit definition of DHE. According to our research, we temporally defined the phenomenon, and the hematoma was significantly absorbed after the occurrence of sICH in closet 14th day after onset, but the PHE apparently aggravated, as delayed perihematomal edema (DHE).^4^ Many studies focus on the hyperacute stage or acute stage after the ictus of sICH, and few recent studies describe DHE. Therefore, our study aims to investigate the risk factors and prognosis of DHE following the sICH and try to demonstrate whether DHE could be a clinical marker of sICH.

## 2 MATERIALS AND METHODS

### 2.1 Clinical data

The retrospective baseline data of 121 patients with sICH (Table 1) who were admitted to the Department of Neurology of the First Affiliated Hospital of Chongqing Medical University from January 2014 to August 2018 were recruited, of whom 38 patients were males and 83 were females, aged from 18 to 86, the median age being 59. All the included 121 patients were diagnosed with sICH in accordance with American Heart Association guidelines by a head computed tomography(CT) examination^2^ within 24 hours after the known time of symptom onset in our hospital, and at least, three more head CT's performed within 24 hours, 5‐9 days, and 12‐20 days after the onset of sICH, retrospectively. All the patients were treated by controlling blood pressure and with conventional dehydration treatment.

**Table 1:** The basic data of DHE group and the non‐DHE group

Variables		Number	Non‐DHE group (85)	DHE group (36)	*P* value
Mean age, years (SD)			62 (52‐71)	52 (43.5‐63)	.002
Sex	Female	83	58 (68.2)	25 (69.4)	.896
	Male	38	27 (31.8)	11 (30.6)	
hospitalization time (d)			19 (16‐24)	25.5 (20‐34.5)	.001
HE boundary	Irregular	37	19 (22.4)	18 (50.0)	.003
	Regular	84	66 (77.7)	18 (50.0)	
Admission BP	SBP		166 ± 22.1	155.3 ± 23.4	.018
	DBP		97.4 ± 14.7	101.3 ± 28.3	.438
Diabetes	No	108	77 (90.6)	31 (86.1)	.525
	Yes	13	8 (9.4)	5 (13.9)	
Hypertension	No	44	28 (32.9)	16 (44.4)	.229
	Yes	77	57 (67.1)	20 (55.6)	
CHD	No	97	66 (77.7)	31 (86.1)	.286
	Yes	24	19 (22.4)	5 (13.9)	
Stroke	No	105	72 (84.7)	33 (91.7)	.388
	Yes	16	13 (15.3)	3 (8.3)	
Smoking	No	75	53 (62.4)	22 (61.1)	.898
	Yes	46	32 (37.7)	14 (38.9)	
Alcohol consumption	No	86	65 (76.8)	21 (58.3)	.044
	Yes	35	20 (23.5)	15 (41.7)	
Location	Lobe	36	25 (29.4)	11 (30.6)	.9
	Basal ganglia	85	60 (70.6)	25 (69.4)	
prothrombin time			12.3 (11.6‐13.1)	12.7 (11.8‐13.9)	.36
fibrinogen level			2.9 (2.4‐3.42)	2.6 (2.2‐3.4)	.268
albumin level			40 (37‐43)	38 (35.5‐40.5)	.061
blood sodium			141.4 ± 3.5	139.7 ± 4.4	.025
Baseline HE volume			7.4 (3.8‐13.5)	19.45 (12.7‐26.1)	<.001

Abbreviations: BP, blood pressure; CHD, Coronary heart disease; DBP, diastolic blood pressure; SBP, systolic blood pressure.

**Figure 1:**
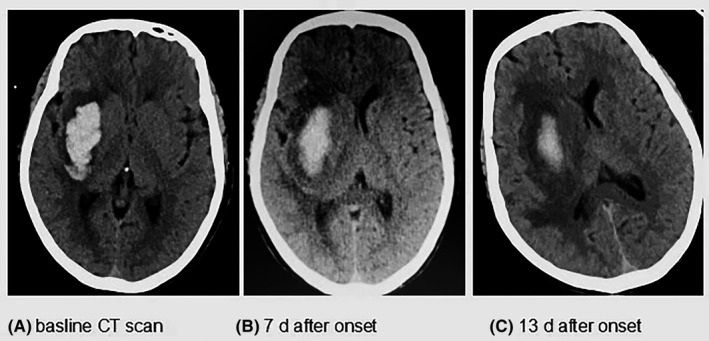
A, describes the sICH on the baseline CT scan within the 24 h after the symptom onset; B, shows the hematoma absorbed and the PHE increased at 7 d after the onset; C, shows us the area of PHE at 14 d is larger than the 7 d PHE but the HE is obviously decreased, named as DHE in our study

We divided these patients into the DHE group and non‐DHE group. The selection criteria of DHE were as follows: head CT obtained within 24 hours, 5‐9 days, 12‐20 days after the onset of sICH. We roughly calculated the volume of HE and PHE based on the CT thresholds. Moreover, the CT value of HE is varying from 44 to 100 Hounsfield units and the PHE is ranging from 5 to 33 Hounsfield units in the head CT. We use the area of every slice on CT multiply the slice thickness and then got the volume of HE and PHE by adding up all results.^2^, ^12^ The volume was blindly calculated by two physicians, and these measurements were used to obtain a volume average which was finally used in the analysis. The volume of PHE minus the volume of hematoma is the volume of absolute PHE. If the volume of absolute PHE in 12‐20 days is 3 mL obviously larger than the volume of absolute PHE in 5‐9 days, this could be defined as DHE in our study (Figure 1).

Of the 121 patients with sICH, 36 patients had DHE shown in the head CT scan, with an incidence of 29.75%. The following lists the exclusion criteria: hematoma expansion, bleeding into ventricles, subarachnoid hemorrhage, other hemorrhage caused by tumor, trauma, cerebral infarction, moyamoya disease, cerebrovascular malformation, or hematological system diseases, and surgical patients. This retrospective and single‐center cohort study did not violate the principles of Declaration of Helsinki and was approved by the ethics committee of the first affiliated hospital of Chongqing medical university [scientific research ethics (2018‐065‐2)].

### 2.2 Methods

Basic, clinically relevant data were collected from sICH patients: gender, age, blood pressure on admission, hospitalization time, hematoma shape, past medical history, the level of blood sodium, baseline hematoma volume within 24 hours after symptom onset, prothrombin time, fibrinogen level, albumin level, the location of hemorrhage, and modified Rankin Scale score (MRS score). As described previously, these factors were used to assess the association with the prognosis of ICH and DHE.

### 2.3 Statistical analysis

Continuous variables that did not satisfy the normal distribution were expressed as median with interquartile range (IQR), and the Mann‐Whitney *U* test was used for comparison between groups. The categorical variables were reported as numbers (n) and percentages of the total (%), and the chi‐square test was used to test the difference between groups. The multivariate logistic regression model was used to screen risk factors of DHE using a stepwise method. The data analysis for this study was generated using SAS 9.4 software (Copyright © 2016 SAS Institute Inc). Significant difference was determined at the *α* level of .05.

## 3 RESULTS

### 3.1 General information

The Table 1 shows us a series of characteristics of basic data between the DHE group and the non‐DHE group of sICH patients. The results of the single factor analysis on each variable determined the differences of age, hospitalization time, the regularity of hematoma border, blood pressure on admission, alcohol consumption, blood sodium levels, and baseline hematoma volume in the two groups were statistically significant (*P* < .05). The other characteristics made no difference between two groups.

### 3.2 Multiple logistic analysis

Variables in which the *P* value < .05 in Table 1, including age, hospitalization time, hematoma shape, blood pressure at admission, alcohol consumption, blood sodium level, baseline hematoma volume, were analyzed for multiple factors of DHE. The results demonstrated that age (OR = 0.958, 95% CI = 0.923‐0.995) and baseline hematoma volume (OR = 1.161, 95% CI = 1.089‐1.238) were the risk factors for sICH patients complicated with DHE which are shown in Table 2.

**Table 2:** Multivariable logistic regression analysis of patients with DHE

Variable	Coefficient	Standard error	Wald *χ* ^2^	*P* value	OR	OR 95% CI
Age	−0.043	0.019	4.946	.026	0.958	0.923‐0.995
Baseline HE volume	0.149	0.033	21.024	<.001	1.161	1.089‐1.238

### 3.3 The prognosis of sICH patients

Whether the variables in Table 1 and the occurrence of DHE influence the prognosis in sICH patients was demonstrated in our study through multiple logistic analysis. The group containing patients' MRS score on discharge ranging from 2 to 6 was founded had a longer hospitalization time, a higher incidence of DHE, a larger hematoma volume, or PHE volume within 24 hours after symptom onset compared with another group containing MRS score within 0‐1, and the difference mentioned between the two groups is statistically significant (*P* value < .05). Meanwhile, the multivariate logistic regression results show only hospitalization time (OR = 1.116, 95% CI = 1.048‐1.188) and the incidence of DHE (OR = 3.062, 95% = 1.196‐7.839) is the significant risk factors of poor prognosis in sICH patients (Table 3).

**Table 3:** Logistic regression analysis of patients with poor prognosis

Clinical characteristics	MRS 0‐1	MRS 2‐6	Univariable logistic analysis		Multivariable logistic analysis^a^	
			*P*‐value	OR (95% CI)	*P*‐value	OR (95% CI)
Hospitalization time	17.5 (15‐23)	24 (20‐29)	.001	4.5 (1.885‐10.744)	.001	1.116 (1.048‐1.188)
Non‐DHE	51 (85.00)	34 (55.74)	<.001	1.135 (1.066‐1.207)	.02	3.062 (1.196‐7.839)
DHE	9 (15.00)	27 (44.26)		1.0 (reference)		1.0 (reference)
Baseline HE volume	6.7 (3.6‐16.6)	12.3 (7.5‐20.8)	.022	1.049 (1.007‐1.092)		
Baseline PHE volume	14.4 (6.85‐24.75)	23.5 (13.9‐35.5)	.008	1.036 (1.009‐1.063)		

a Stepwise method is used to screen variables

## 4 DISCUSSION

Our study demonstrates that the logistic analysis of all possible factors in Table 1 concluded that DHE and hospitalization time were indeed risk factors affecting the prognosis of sICH patients. The longer the hospitalization time was, the worse the prognosis of sICH patients was, which may be due to the illness itself. Delayed perihematomal edema is likely to be a clinical indicator for poor prognosis or a higher MRS score in our study, of which proves to be a problem that clinicians should be highly aware. Prior study in 2016 identified that PHE volume increases with time,^2^ and increasing studies founded that PHE is responsible for the prognosis of sICH patients,^3^, ^13^, ^14^ as well as DHE.^15^ Hence, it is quite prerequisite to analyze the possible risk factors that may lead to DHE so that physicians could take better treatment as early as possible in order to reduce the subsequent deterioration and improve patients' functional outcomes. Results from our patients in the DHE multifactor analysis study showed that initial hematoma volume and the effects of age are risk factors for DHE after sICH, concluding that the larger the initial hematoma volume, the more prone to DHE, which is in accordance with the results of previous studies.^7^, ^8^, ^16^ A recent review article detailed that a few days or weeks after sICH, the main cause of PHE is the release of hemoglobin degradation products after the red blood cells are dissolved; perhaps according to larger cerebral hemorrhage initial hematoma volume, the greater the toxicant secondary to the hemoglobin degradation more prone to cause the DHE.^4^ Inflammation post‐ICH, acknowledged by many researchers, plays a critical role in the breakdown of the blood brain barrier (BBB) and the late formation of PHE.^5^, ^6^, ^17^ An in vivo study by SHIZHOU Lin and colleagues identified that leonurine could ameliorate PHE through attenuating BBB breakdown, reducing the inflammatory response and decreasing the amount of hemoglobin degradation, which maybe support our results to some extent.^5^ The larger baseline hematoma volume triggers a more severe inflammatory response and leads to the extensive damage of BBB which conspire to create the DHE. A study by H. Arima and colleagues found that the baseline hematoma volume is the main factor of PHE,^18^, ^19^ so it is impossible to ignore the influence of initial hematoma volume on the development of DHE. On the other hand, in ICH patients, the hematoma itself can lead to the increase in ICP, and the increased ICP causes a decrease in cerebral blood flow and cerebral perfusion pressure around the hematoma; the continuous decrease in cerebral blood flow and cerebral perfusion pressure around the hematoma creates an ischemic area around the hematoma, resulting in the damage of the blood brain barrier and the increase in vasogenic edema, which supports our conclusion.^10^, ^20^

In addition, our study demonstrated that increased age decreases the risk for DHE. It may be that with increased age, there is more brain atrophy, decreasing the odds for DHE; clinicians should pay close attention to younger patients with sICH as they are more likely to develop DHE. But the specific mechanism is not well understood. A recent systematic review demonstrated that the growth of PHE was a significant predictor of ICH in the short term and long term.^20^ International studies place more emphasis on the study of PHE in the super‐acute stage and the acute stage of sICH. Few studies research the occurrence of DHE after sICH and its pathophysiology.

Due to our retrospective design, not every patient met our criteria of having a timely head CT scan, causing the small sample size and limiting statistical power. We recommend future prospective studies and animal experiments to identify and explain this phenomenon. Presently, this study suggests that DHE is likely to affect the prognosis of patients with sICH, and therefore, clinicians should be aware of DHE. Future studies should further current knowledge on DHE, especially regarding its role in late‐onset brain edema.

## 5 CONCLUSION

Our data define the DHE and conclude that the occurrence of DHE is closely related to the poor prognosis of sICH patients. We suggest that clinicians be aware and alert of these factors. It is necessary for clinicians to repeat the head CT scan in 2 weeks or even a few weeks after the onset of sICH, especially those with symptoms like vomiting and headache. Otherwise, our research also suggests that the baseline hematoma volume and age are risk factors of DHE. Considering the impacts of DHE, we propose with larger initial hematoma volume, the early operation should be more aggressive, and the dehydration treatment should be strengthened in clinical work to minimize the risk of DHE and improve the prognosis of DHE. For younger sICH patients, observation of their condition should also be closely and timely, so as to timely adjust the targeting treatment and reduce the incidence of DHE.

## CONFLICTS OF INTEREST

The authors declared no potential conflicts of interest with respect to the research, authorship, and/or publication of this article. Informed Consent: Informed consent was obtained from all individual participants included in the study.

## ETHICAL APPROVAL

This retrospective and single‐center cohort study did not violate the principles of Declaration of Helsinki and was approved by the ethics committee of The First Affiliated Hospital of Chongqing Medical University [scientific research ethics (2018‐065‐2)]. All persons gave their informed consent prior to our study.
